# Adaptive leadership, organizational culture and patient-perceived service quality in private healthcare: evidence from Addis Ababa

**DOI:** 10.1108/IJHCQA-09-2025-0136

**Published:** 2025-11-14

**Authors:** Biniam Ali Eshete, Tilaye Kassahun

**Affiliations:** Graduate School of Business Leadership, University of South Africa, Pretoria, South Africa; Department of Business Management, St. Mary’s University, Addis Ababa, Ethiopia

**Keywords:** Adaptive leadership, Organizational culture, Patient-perceived service quality, Private healthcare, Ethiopia, PLS-SEM

## Abstract

**Purpose:**

This study investigates how adaptive leadership and organizational culture influence patient-perceived service quality in private healthcare institutions in Addis Ababa, Ethiopia. It further examines the mediating role of organizational culture in the relationship between leadership and service quality.

**Design/methodology/approach:**

A quantitative, cross-sectional survey design was employed with data collected from 355 patients across 14 private hospitals. Established scales for adaptive leadership, organizational culture and service quality (SERVQUAL) were used. Data were analyzed using partial least squares structural equation modeling (PLS-SEM) to test direct, indirect and mediating effects.

**Findings:**

The results show that adaptive leadership has a significant positive effect on both organizational culture and patient-perceived service quality. Organizational culture also directly enhances service quality and partially mediates the relationship between adaptive leadership and service quality. The model explained 45.4% of the variance in service quality, highlighting the critical role of intangible organizational factors in shaping patient experiences.

**Practical implications:**

The findings suggest that private healthcare providers should invest in leadership development programs that strengthen adaptive leadership capabilities and foster supportive, patient-centered cultures. Policymakers and managers should recognize that improving service quality requires not only technical and infrastructural investments but also attention to leadership and cultural dynamics.

**Originality/value:**

This study contributes to the literature by extending adaptive leadership and organizational culture research into the underexplored context of Sub-Saharan Africa’s private healthcare sector. It provides empirical evidence of the mediating role of culture, offering theoretical and practical insights into how organizational resources shape patient experiences.

## 1. Introduction

Patient experience is widely recognized as a core dimension of healthcare quality alongside clinical effectiveness and safety, and it has been shown to influence adherence, outcomes, and safety indicators (Oben, 2020). In urban contexts, private healthcare service providers play an increasingly important role in meeting demand, with patient-perceived service quality shaping trust, satisfaction, and loyalty (Nguyen *et al*., 2021). Beyond infrastructure and technical skill, organizational factors such as leadership and culture significantly shape patient interactions and outcomes with healthcare service providers (Huang *et al*., 2024).

Despite this recognition, there is little empirical research in Sub-Saharan Africa that integrates leadership and organizational culture to explain patient-perceived service quality. Most studies are based on high-income contexts, with limited transferability to African healthcare systems characterized by resource constraints, institutional complexity, and rapid private sector expansion (Johnson *et al*., 2021; Nwankpa and Datta, 2021). Existing evidence often treats leadership and culture separately, while recent reviews emphasize the need for studies examining their combined and mediating effects on patient perceptions (Ahmed *et al*., 2024; Huang *et al*., 2024). In Ethiopia, the role of these intangible organizational factors within the private sector remains especially underexplored, despite evidence that patient experiences are increasingly tied to performance and equity (Siddiqi *et al*., 2023).

The present study examines the influence of adaptive leadership and organizational culture on patient-perceived service quality in Addis Ababa’s private healthcare sector. Adaptive leadership is relevant for healthcare because of its focus on flexibility, mobilizing people, and problem-solving in complex systems (Laur *et al*., 2021). Organizational culture, understood as shared values, beliefs, and norms, has been consistently linked to quality and safety outcomes (Aven and Ylönen, 2021; Panda and Haynes, 2020). This study applies a quantitative design using patient survey data and structural equation modeling to assess both direct and mediated effects, consistent with recent methodological calls in healthcare quality research.

This study contributes to theory and practice in three ways. First, it extends adaptive leadership and organizational culture scholarship to a Sub-Saharan African private healthcare context, where empirical studies are limited. Much of the existing literature on patient-perceived service quality, leadership, and organizational culture has been developed in high-income countries with more stable resources and different cultural expectations. In contrast, Sub-Saharan Africa and Ethiopia in particular presents a healthcare environment marked by resource constraints, rapidly expanding private institutions, and unique socio-cultural dynamics. By addressing this gap, the present study advances leadership and service quality research in the underexplored private healthcare sector of Addis Ababa, offering insights relevant to low- and middle-income countries (LMICs).

Second, it empirically tests the mediating role of organizational culture between leadership and patient-perceived service quality, responding to gaps highlighted in recent reviews.

Third, it provides practical insights for managers and policymakers by demonstrating how leadership practices and cultural orientations can be leveraged to strengthen patient experiences, reinforcing evidence that organizational factors are as important as technical capacity in shaping healthcare quality.

To examine how adaptive leadership and organizational culture influence patient-perceived service quality in Addis Ababa’s private healthcare sector, and to test whether organizational culture mediates the relationship between leadership and service quality.

1. Does adaptive leadership directly improve patient-perceived service quality?
2. To what extent does organizational culture influence patient-perceived service quality?
3. Does organizational culture mediate the relationship between adaptive leadership and patient-perceived service quality?

## 2. Literature review

### 2.1 Theoretical framework

This study is grounded in an integrative framework combining Adaptive Leadership Theory, the Resource-Based View (RBV), and the SERVQUAL model. Adaptive Leadership Theory highlights leaders’ ability to mobilize organizations in complex, changing environments particularly relevant in resource-constrained healthcare. RBV frames leadership and organizational culture as strategic intangible resources that generate sustained advantage. SERVQUAL operationalizes patient perceptions of service delivery, linking internal organizational dynamics to external outcomes. Together, these perspectives position adaptive leadership as a driver of organizational culture (RBV), which in turn shapes patient-perceived service quality (SERVQUAL), forming a robust foundation for hypothesis development.

#### 2.1.1 Adaptive leadership theory

Adaptive leadership theory emphasizes the capacity of leaders to mobilize employees, facilitate learning, and respond to complex, systemic challenges (Heifetz *et al*., 2009). In contrast to transactional or authoritarian approaches, adaptive leadership does not rely on positional authority or prescriptive decision-making but instead fosters distributed problem-solving and employee engagement (Uhl-Bien and Arena, 2018). Within healthcare, where patient needs, clinical technologies, and regulatory requirements evolve continuously, adaptive leadership provides a mechanism for building resilience and innovation (Wiig *et al*., 2020). This theoretical lens explains how leaders encourage frontline staff to experiment, challenge existing practices, and collaborate in the pursuit of patient-centered solutions. By creating an environment of trust and psychological safety, adaptive leaders enable healthcare professionals to manage uncertainty and deliver high-quality patient care.

#### 2.1.2 Resource-Based View (RBV) of the firm

The RBV posits that sustainable organizational advantage derives from resources that are valuable, rare, inimitable, and non-substitutable (VRIN) (Barney, 1991). Leadership capabilities and organizational culture can be conceptualized as intangible strategic resources that meet these criteria. Adaptive leadership represents a rare and socially complex capability that is difficult to replicate across organizations, while a strong patient-centered culture constitutes a path-dependent asset that evolves over time through shared norms and practices (Laur *et al*., 2021). When effectively developed, these internal resources enhance coordination, foster staff commitment, and align organizational processes with patient expectations, thereby yielding superior service outcomes (House *et al*., 2021). RBV therefore provides a theoretical basis for conceptualizing leadership and culture as complementary drivers of service quality within healthcare institutions.

#### 2.1.3 SERVQUAL framework

Service quality theory, particularly the SERVQUAL framework, emphasizes that the quality of services is determined by the extent to which performance meets or exceeds customer expectations across dimensions such as reliability, responsiveness, assurance, empathy, and tangibles (Parasuraman *et al.*, 1988). In the healthcare sector, these dimensions translate into patient-centered care practices, effective communication, and compassionate interactions that directly shape patient experiences. Service quality has been a central theme in both management and healthcare research. Raza *et al*. (2020), applying a modified e-SERVQUAL model in Internet banking, showed that service quality significantly enhances customer satisfaction and loyalty. Although their study was conducted in the financial sector, the findings underscore a universal principle: service quality is a key determinant of client satisfaction and loyalty, a principle equally relevant in healthcare. Leadership and culture play a central role in narrowing the gap between patient expectations and perceptions by embedding quality-oriented norms into clinical and administrative routines (Huang *et al*., 2024). Adaptive leadership, supported by a positive organizational culture, ensures that patient-facing processes are designed to consistently deliver reliability, empathy, and responsiveness, thereby enhancing overall service quality.

By integrating adaptive leadership theory, RBV, and service quality theory, this study proposes a holistic model where leadership behaviors and organizational culture function as interdependent resources that shape patient-perceived service quality. Adaptive leadership fosters conditions of openness, innovation, and learning, which in turn cultivate a supportive organizational culture. This culture acts as the conduit through which leadership values and practices are institutionalized, ultimately narrowing the service quality gap. Framing leadership and culture as VRIN resources underscores their strategic importance in sustaining superior patient experiences and outcomes. This theoretical framework thus provides a strong foundation for examining the hypothesized direct, indirect, and mediating relationships among adaptive leadership, organizational culture, and patient-perceived service quality.

### 2.2 Adaptive leadership and patient-perceived service

Adaptive leadership has emerged as a critical approach to addressing the complexities of contemporary healthcare systems. Defined as the capacity to mobilize people, encourage innovation, and respond effectively to challenges through learning and adaptation (Heifetz *et al*., 2009), adaptive leadership differs from transactional or authoritarian approaches, which emphasize command and compliance. Instead, adaptive leaders foster collaboration, openness to change, and the resolution of value conflicts. These qualities are particularly important in healthcare, where evolving patient needs, rapid technological advancements, and persistent systemic challenges such as resource constraints and patient safety require innovative solutions.

Rather than providing all the answers, adaptive leaders create environments where staff are empowered to identify challenges, experiment with solutions, and collaborate across disciplines. Empirical studies have shown that adaptive leadership enhances staff engagement, improves teamwork, reduces burnout, and strengthens clinical outcomes (Uhl-Bien and Arena, 2018). More recent findings further highlight its role in fostering resilience and psychological safety during health crises, ultimately enhancing the quality of patient care (Rangachari and Woods, 2020). Based on this evidence, it is expected that adaptive leadership directly enhances how patients perceive the quality of healthcare services.

H1.
Adaptive leadership has a positive effect on patient-perceived service quality.

### 2.3 Organizational culture and service quality

Organizational culture refers to the shared values, beliefs, and assumptions that guide behavior and decision-making within an organization (Fernandes *et al*., 2023). In healthcare, culture plays a decisive role in shaping both organizational performance and patient outcomes. A culture grounded in compassion, accountability, collaboration, and patient-centeredness has consistently been linked with high-quality service delivery and improved patient outcomes (Macgillivray, 2020; Scane *et al*., 2022). In healthcare, leadership and workplace dynamics play a crucial role in staff satisfaction and service delivery. Shah *et al*. (2018) identified job clarity, compensation, supervisor support, and empowerment as key drivers of nurse satisfaction, while Dahri *et al*. (2019) highlighted the negative impact of despotic leadership and occupational stress, which reduce satisfaction through burnout. Workplace incivility further harms staff well-being and job satisfaction (Dahri and Ab Hamid, 2018), whereas resonant leadership can buffer these effects (Dahri *et al.*, 2020).

Positive cultures encourage effective communication, reduce errors, and foster collaboration, which directly shape patients’ experiences and perceptions of service quality (Macgillivray, 2020). When patients perceive that care environments reflect empathy, responsiveness, and accountability, they report higher satisfaction with service quality. This underscores the importance of culture as a critical organizational resource.

H2.
Organizational culture has a positive effect on patient-perceived service quality.

### 2.4 Adaptive leadership and organizational culture

Leadership and culture are inherently interconnected, with leaders often seen as the primary architects of organizational culture. Through their behaviors, values, and priorities, leaders shape the norms and expectations that guide organizational practices (Shanafelt *et al*., 2021). Adaptive leaders, in particular, foster cultures of openness, experimentation, and continuous learning attributes essential for agility in dynamic healthcare contexts (Laur *et al*., 2021). By modeling collaboration and accountability, they embed patient-centeredness into organizational routines, influencing how services are ultimately delivered.

As such, adaptive leadership is expected to positively shape organizational culture by embedding values that support collaboration, accountability, and patient-centered care.

H3.
Adaptive leadership has a positive effect on organizational culture.

### 2.5 The mediating role of organizational culture

While adaptive leadership directly influences both patient-perceived service quality and organizational culture, culture itself serves as an essential pathway through which leadership translates into improved outcomes (Tate *et al*., 2023). Leaders create the values and norms that guide staff behaviors, but it is the culture that operationalizes these behaviors into daily practices that affect patient experiences.

Recent scholarship emphasizes this mediating role, showing that organizational culture often acts as the mechanism connecting leadership practices with performance outcomes in healthcare (Mohammed and Al-Abrrow, 2022; Tate *et al*., 2023). In this sense, adaptive leadership nurtures a culture of accountability, collaboration, and patient-centeredness, which in turn drives patients’ perceptions of service quality. Therefore, it is expected that organizational culture will mediate the relationship between adaptive leadership and patient-perceived service quality.

H4.
Organizational culture mediates the relationship between adaptive leadership and patient-perceived service quality.

**Figure 1:**
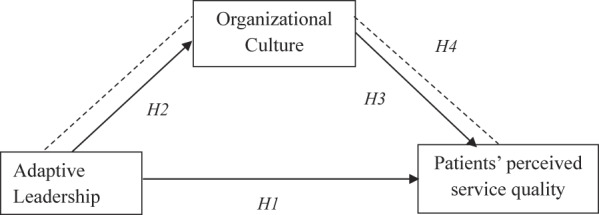
Conceptual framework of the study. Source: Authors’ own work

*Based on the hypothesized relationships among the variables, the following conceptual framework was constructed, as depicted in* Figure 1.

## 3. Methodology

This study employed a quantitative, cross-sectional research design to examine the relationships between adaptive leadership, organizational culture, and patient-perceived service quality in private healthcare settings. The target population consisted of patients who had recently received care in 14 private hospitals in Addis Ababa. Patients were selected from 14 private hospitals in Addis Ababa using stratified sampling based on hospital size (bed capacity) to capture variation across small, medium, and large institutions. This approach reflects the diverse structure of Ethiopia’s private healthcare sector, where hospitals differ substantially in scale and resources. Within each stratum, patients were randomly approached in waiting areas, yielding 355 usable responses. The sample size exceeds the PLS-SEM “10-times rule” requirement and the 200 minimum for the study’s model complexity, ensuring adequate statistical power and reliable estimates (Hair *et al*., 2021). The instrument was a structured questionnaire adapted from validated scales: adaptive leadership items from Heifetz *et al*. (2009), organizational culture items from Schein (2010), and patient-perceived service quality items based on the SERVQUAL framework (Parasuraman *et al.*, 1988). Ethical approval for this study was granted by the University of South Africa, Graduate School of Business Leadership Research Ethics Review Committee (UNISA SBL RERC). Responses were collected on a five-point Likert scale, ranging from strongly disagrees to strongly agree Table 1.

**Table 1:** Table measurement items

Construct	Dimension	Item	Statement	Source
Adaptive Leadership	GWP – Giving Work Back to People	GWP1	Hospital leaders empower staff to make independent decisions	Heifetz *et al*. (2009)
		GWP2	Staff are encouraged to take responsibility for patient care	
		GWP3	Leaders provide guidance but allow staff to solve problems on their own	
		GWP4	Staff are trusted to handle complex patient care situations	
	IACs – Identifying Adaptive Challenges	IAC1	Leaders recognize complex issues affecting patient care	
		IAC2	The hospital identifies challenges that require creative solutions	
		IAC3	Leaders clearly communicate service challenges to staff	
		IAC4	Staffs are aware of the organization’s critical problems	
		IAC5	Adaptive challenges are addressed proactively by leadership	
	MDA – Mobilizing for Adaptive Work	MDA1	Leaders motivate staff to work collaboratively to solve problems	
		MDA2	Staffs are encouraged to participate in improving hospital services	
		MDA3	Leadership inspires staff to adapt to changing patient needs	
		MDA4	Leaders effectively coordinate teams for problem-solving	
		MDA5	Staffs are actively involved in implementing improvements	
	RD – Regulating Distress	RD1	Leaders manage stressful situations calmly	
		RD2	Staffs provide support to patients during challenging moments	
		RD3	Leaders maintain focus and discipline during crises	
		RD4	Staffs handle patient complaints and emergencies effectively	
		RD5	The hospital environment helps patients feel safe and supported	
C. Service Quality (SERVQUAL)	E − Empathy	E1	Staff gives patients individual attention	Parasuraman *et al.* (1988)
		E2	Staff understands the specific needs of each patient	
		E3	Hospital staffs are caring and considerate	
	R – Reliability	R1	Services are provided as promised	
		R2	Appointments and schedules are reliable	
		R3	Staff performs tasks accurately and consistently	
	RE – Responsiveness	RE1	Staff responds promptly to patient requests	
		RE2	Staffs are willing to help whenever needed	
		RE3	Patients receive timely information about services	
	T – Tangibles	T1	Hospital facilities are modern, clean, and well-maintained	
		T2	Medical equipment is up-to-date and functional	
		T3	Staff appearance is professional and neat	
Organizational Culture (OC)	OC	OC1	The hospital encourages teamwork and collaboration	Schein (2010)
		OC2	Staff shares common values prioritizing patient care	
		OC3	The hospital promotes continuous improvement in services	
		OC4	Leadership fosters a culture of accountability and responsibility	

**Source(s):** Authors’ own work

Data analysis was conducted using Partial Least Squares Structural Equation Modeling (PLS-SEM) with SmartPLS, chosen for its suitability in handling complex models with latent constructs and predictive orientation. Reliability and validity were assessed through Cronbach’s alpha, composite reliability, and average variance extracted (AVE). Discriminant validity was evaluated using the Fornell–Larcker criterion and HTMT ratios. The structural model was then tested to examine the hypothesized direct, indirect, and mediating effects of organizational culture. Bootstrapping with 5,000 resamples was applied to assess the significance of path coefficients.

## 4. Result

### 4.1 Demographic profile of respondents

The target respondents of this study were patients of a private hospital. As indicated in Table 2 the majority were aged 31–40 years (44.8%), followed by those aged 51 years and above (24.2%), while smaller proportions were within the 41–50 years (18.0%) and 18–30 years (13.0%) categories. In terms of gender, males accounted for 54.1% and females 45.9%, indicating a fairly balanced distribution. Educational attainment was dominated by Diploma holders (38.6%) and BSc/BA degree holders (38.0%), with fewer respondents having MSc/MA qualifications (5.9%) or other forms of education (17.5%). With respect to service utilization, the highest proportion of respondents visited the Outpatient Department (35.2%), followed by Inpatient services (25.9%), Laboratory (12.4%), MCH (10.7%), Pharmacy (8.5%), and other departments (7.3%). Regarding the length of stay, the majority reported staying for days (60.6%), while 26.8% stayed for a week, 9.6% for a month, and only 3.1% remained for more than two months.

**Table 2:** Profile of respondents

Variable	Frequency	Percent
Age		
18–30	46	13.0%
31–40	159	44.8%
41–50	64	18.0%
51+	86	24.2%
Gender		
Male	192	54.1%
Female	163	45.9%
Education		
Diploma	137	38.6%
BSc/BA	135	38.0%
MSc/MA	21	5.9%
Other	62	17.5%
Department Visited		
Laboratory	44	12.4%
Pharmacy	30	8.5%
OPD	125	35.2%
MCH	38	10.7%
Inpatient	92	25.9%
Other	26	7.3%
Continuous Stay		
For days	215	60.6%
For a week	95	26.8%
For a month	34	9.6%
Above 2 months	11	3.1%

**Source(s):** Authors’ own work

### 4.2 Measurement model assessment

#### 4.2.1 Construct reliability and validity

**Figure 2:**
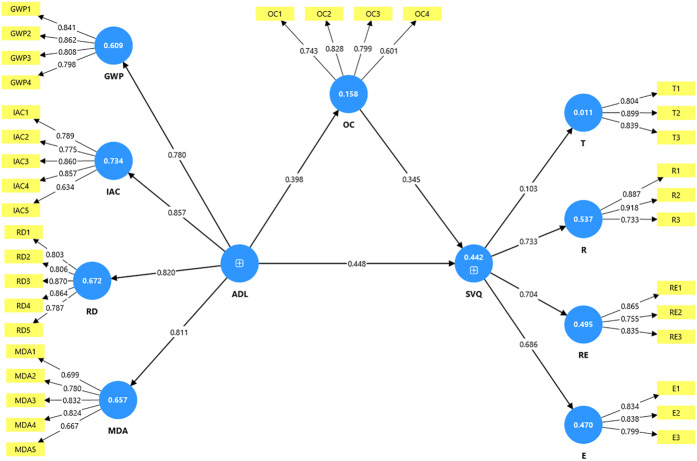
Measurement model. Source: Authors’ own work

The results Table 3 indicate that all constructs demonstrate acceptable levels of reliability and validity. Cronbach’s alpha values range from 0.736 to 0.884, exceeding the recommended threshold of 0.70, which shows good internal consistency. Similarly, both composite reliability (rho_a and rho_c) values are above 0.70 for all constructs, confirming strong reliability. In terms of convergent validity, the average variance extracted (AVE) values range from 0.560 to 0.722, with all exceeding the minimum acceptable level of 0.50, indicating that each construct explains more than half of the variance of its indicators. Overall, the measures used in this study meet the standard criteria for construct reliability and validity Figure 2.

**Table 3:** Construct reliability and validity

	Cronbach’s alpha	Composite reliability (rho_a)	Composite reliability (rho_c)	Average variance extracted (AVE)
E	0.763	0.765	0.864	0.679
GWP	0.846	0.847	0.897	0.685
IAC	0.842	0.844	0.890	0.620
MDA	0.818	0.825	0.874	0.583
OC	0.736	0.723	0.834	0.560
R	0.803	0.817	0.886	0.722
RD	0.884	0.890	0.915	0.684
RE	0.754	0.762	0.859	0.672
T	0.812	0.879	0.885	0.720

**Source(s):** Authors’ own work

Table 4 presents the results of discriminant validity using both the HTMT ratio and the Fornell-Larcker criterion. The HTMT values are all below the conservative threshold of 0.85, suggesting that the constructs are empirically distinct from one another. Likewise, the Fornell-Larcker results. Source: Authors’ own work.

**Table 4:** Discriminant validity HTMT

	E	GWP	IAC	MDA	OC	R	RD	RE	SVQ	T
E										
GWP	0.464									
IAC	0.516	0.656								
MDA	0.380	0.750	0.644							
OC	0.633	0.494	0.395	0.377						
R	0.300	0.448	0.344	0.407	0.434					
RD	0.424	0.481	0.793	0.605	0.308	0.332				
RE	0.299	0.587	0.310	0.668	0.292	0.380	0.282			
T	0.115	0.098	0.226	0.161	0.276	0.032	0.198	0.214	0.677	

**Source(s):** Authors’ own work

Table 5 confirm discriminant validity, as the square roots of the AVEs (diagonal values) are greater than the correlations between constructs (off-diagonal values). For example, the square root of AVE for E (0.824) is higher than its correlations with other constructs, and a similar pattern is observed across all constructs. Together, these findings provide strong evidence that the constructs used in the study is both reliable and distinct, satisfying the requirements for discriminant validity.

**Table 5:** Discriminant validity: Fornell-Larcker

	E	GWP	IAC	MDA	OC	R	RD	RE	T
E	0.824								
GWP	0.372	0.828							
IAC	0.415	0.554	0.787						
MDA	0.302	0.620	0.537	0.763					
OC	0.522	0.403	0.326	0.305	0.748				
R	0.235	0.361	0.279	0.329	0.325	0.850			
RD	0.355	0.427	0.686	0.521	0.272	0.275	0.827		
RE	0.228	0.472	0.246	0.528	0.234	0.299	0.232	0.820	
T	0.040	0.051	−0.173	−0.072	0.220	−0.009	−0.165	0.057	0.848

Source: Authors’ own work

#### 4.2.2 Outer loadings for adaptive leadership (second-order construct)

Table 6 shows the outer loadings of the indicators on their respective construct (ADL). All factor loadings exceed the recommended threshold of 0.70, ranging from 0.770 (RD) to 0.832 (MDA), which indicates strong indicator reliability. The t-statistics are well above the critical value of 1.96, and all *p*-values are 0.000, confirming that the loadings are statistically significant. These results demonstrate that each indicator contributes meaningfully to the measurement of Adaptive Leadership (ADL), providing evidence of strong convergent validity at the indicator level.

**Table 6:** Outer loadings for adaptive leadership (second-order construct)

	Original sample (O)	Outer loading	Standard deviation (STDEV)	T Statistics (|O/STDEV|)	*p* Values
GWP ← ADL	0.829	0.828	0.022	37.031	0.000
IAC ← ADL	0.831	0.828	0.023	36.228	0.000
MDA ← ADL	0.832	0.831	0.021	40.357	0.000
RD ← ADL	0.770	0.768	0.037	21.012	0.000

**Source(s):** Authors’ own work

### 4.3 Structural model assessment

The results in Table 7 indicate that the model has acceptable explanatory power and predictive relevance. The *R*^2^ values show that Adaptive Leadership explains 16.5% of the variance in Organizational Culture (OC) and, together with OC, explains 45.4% of the variance in Service Quality (SVQ). The *f*^2^ values suggest that Adaptive Leadership has a small-to-medium effect on OC (0.198) and a medium effect on SVQ (0.331), while OC has a small-to-medium effect on SVQ (0.170). The *Q*^2^ values are above zero for both OC (0.157) and SVQ (0.355), indicating that the model has predictive relevance for these constructs. Overall, the findings confirm that Adaptive Leadership and Organizational Culture play meaningful roles in shaping Service Quality.

**Table 7:** Structural Model Assessment (R2, f2, and Q2)

Metric	OC	SVQ	ADL → OC	ADL → SVQ	OC → SVQ
*R* ^2^	0.165 (16.5%	0.454 (45.4%	–	–	–
*f* ^2^	–	–	0.198	0.331	0.170
*Q* ^2^	0.157	0.355	–	–	–

**Source(s):** Authors’ own work

**Figure 3:**
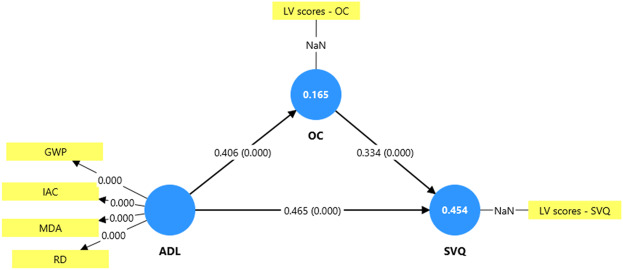
Structural model. Source: Authors’ own work

The results in Table 8 show that both direct and indirect effects in the model are statistically significant. For the direct paths, Adaptive Leadership (ADL) has a strong positive effect on OC (*β* = 0.406, *t* = 6.974, *p* < 0.001) and on Service Quality (SVQ) (*β* = 0.465, *t* = 10.665, *p* < 0.001). Additionally, OC significantly influences SVQ (*β* = 0.334, *t* = 7.083, *p* < 0.001). Regarding the indirect effect, ADL also affects SVQ through OC (*β* = 0.136, *t* = 4.428, *p* < 0.001), indicating partial mediation. These findings confirm that ADL enhances SVQ both directly and indirectly, with OC acting as a significant mediator in the relationship Figure 3.

**Table 8:** Hypothesis testing

Hypothesis	Path	Original sample (O)	*T* statistics	*p* Values	Decision
Direct effect					
H1	ADL → SVQ	0.465	10.665	0.000	Supported
H2	ADL → OC	0.406	6.974	0.000	Supported
H3	OC → SVQ	0.334	7.083	0.000	Supported
Indirect effect					
H4	ADL → OC → SVQ	0.136	4.428	0.000	Supported

**Source(s):** Authors’ own work

## 5. Discussion

This study examined the relationships between Adaptive Leadership (ADL), Organizational Culture (OC), and Patient-Perceived Service Quality (SVQ) in private healthcare settings in Addis Ababa. The results provide evidence that adaptive leadership significantly influences both organizational culture and service quality, highlighting its critical role in healthcare management. Specifically, ADL had a positive and significant direct effect on OC (*β* = 0.406, *p* < 0.001), indicating that leaders who demonstrate flexibility, responsiveness, and the ability to mobilize staff foster a supportive and performance-oriented organizational culture. This aligns with (Heifetz, 1994) Adaptive Leadership Theory, which emphasizes the role of leaders in guiding organizations through complex and dynamic environments, such as healthcare, by encouraging learning, collaboration, and innovation.

The study also found a significant direct effect of ADL on patient-perceived service quality (*β* = 0.465, *p* < 0.001), suggesting that adaptive leaders positively shape patients’ perceptions by promoting effective processes, staff engagement, and responsiveness to patient needs. This supports prior research indicating that leadership behaviors in healthcare directly affect service delivery outcomes (Elkomy *et al*., 2023; Restivo *et al*., 2022). Furthermore, the findings show that OC significantly influences SVQ (*β* = 0.334, *p* < 0.001), demonstrating that a strong, positive organizational culture enhances service quality by reinforcing shared values, teamwork, and patient-centered practices. This is consistent with the literature emphasizing that organizational culture serves as a critical mechanism through which leadership translates into performance outcomes in healthcare (ALFadhalah and Elamir, 2021; Bernardes *et al*., 2020).

Importantly, the mediation analysis revealed that OC partially mediates the relationship between ADL and SVQ (indirect effect *β* = 0.136, *p* < 0.001). This indicates that adaptive leaders improve service quality not only through direct actions but also by shaping an organizational culture that supports patient-focused behaviors and practices. The findings align with previous studies that suggest leadership affects organizational outcomes both directly and indirectly through cultural mechanisms (Kucharska, 2021; Lokaj and Sadrija, 2020).

The present study’s finding that adaptive leadership enhances both organizational culture and patient-perceived service quality resonates with prior evidence linking leadership to performance outcomes. Similar to Shah *et al*. (2018), who demonstrated that empowerment and compensation improve nurse satisfaction, our results highlight the importance of supportive organizational dynamics in fostering positive patient experiences. In line with Dahri *et al*. (2019), we also affirm that leadership style directly affects workplace outcomes; however, unlike despotic leadership’s negative impact on job satisfaction, adaptive leadership cultivates a constructive culture that enhances service quality.

Moreover, our findings regarding the mediating role of organizational culture mirror Samad Dahri *et al*. (2020) insights: workplace culture and emotional climate shape the relationship between leadership and satisfaction-related outcomes. While their work identified resonant leadership as a buffer against incivility and burnout, our study extends this by showing that adaptive leadership not only mitigates negative workplace effects but also proactively strengthens organizational culture, which patients perceive through improved service quality.

Finally, although Raza *et al*. (2020) investigated service quality in Internet banking, their conclusion that satisfaction drives loyalty parallels our findings in healthcare. Just as e-SERVQUAL dimensions foster satisfaction and loyalty in online banking, adaptive leadership and supportive culture foster satisfaction and loyalty in healthcare services. This suggests that the service quality satisfaction loyalty nexus may be robust across industries, reaffirming the centrality of intangible organizational factors in shaping client experiences.

Overall, these results highlight the central role of adaptive leadership in private healthcare settings. Leaders who are responsive, supportive, and able to manage change effectively can cultivate a positive organizational culture, which in turn enhances patient-perceived service quality. For healthcare administrators and policymakers in Addis Ababa, these findings underscore the importance of leadership development programs that strengthen adaptive leadership competencies and promote a culture conducive to high-quality patient care.

## 6. Implications for theory

The findings of this study make a significant and unique contribution to the existing body of knowledge in leadership and healthcare management. First, our research provides robust empirical evidence supporting Heifetz’s (1994) Adaptive Leadership Theory in a novel and underrepresented context private healthcare institution in a developing country. While previous studies have largely validated this theory in Western, public-sector settings, our results demonstrate that adaptive leadership behaviors are equally critical for navigating complex challenges in a rapidly evolving, market-driven healthcare environment in Africa. This contextual validation extends the theory’s applicability beyond its traditional scope and underscores its universality.

Furthermore, this study provides a granular understanding of the mechanisms through which adaptive leadership influences organizational outcomes. By confirming the partial mediation of organizational culture, our findings reinforce the theoretical proposition that culture is not merely a byproduct but a critical conduit for leadership influence. This suggests that adaptive leaders do not simply command change; they first cultivate a cultural environment of psychological safety, collaboration, and learning, which in turn facilitates the successful implementation of new strategies and behaviors. This finding enriches the existing literature by specifying the pathway from leadership style to organizational effectiveness, providing a more nuanced theoretical model for future research.

Finally, our research extends the conceptual understanding of “service quality” in the context of private healthcare. By linking specific leadership behaviors and organizational culture to patient-perceived service quality, we move beyond a purely technical or clinical definition of quality. The study suggests that service quality is a holistic construct, deeply influenced by the cultural and interpersonal dynamics within the institution. This theoretical refinement offers a new lens for academics studying service management, encouraging future research to integrate organizational and cultural variables into their models of service quality.

## 7. Implications for practice

The results of this study offer direct and actionable insights for healthcare administrators, managers, and policymakers, particularly in contexts similar to Addis Ababa. The primary implication is the need to prioritize adaptive leadership as a core element of management training. Leaders who can diagnose systemic challenges, mobilize staff, and create safe spaces for collaborative problem-solving are better positioned to enhance service delivery. Accordingly, healthcare organizations should invest in leadership development programs that build competencies in emotional intelligence, systems thinking, and facilitation, extending beyond traditional management techniques.

Equally important is the cultivation of a positive and resilient organizational culture. The findings indicate that cultures characterized by flexibility, openness to change, and commitment to patient-centered care are powerful drivers of service quality. Managers can reinforce this by fostering participative decision-making, promoting learning from failure, and aligning employee values with organizational goals. Practical initiatives such as cross-functional teamwork, structured feedback systems, and recognition programs that reward collaboration can embed these values in everyday operations.

For policymakers and institutional leaders in developing countries, the study underscores that improving healthcare quality requires more than infrastructure and technology upgrades. Targeted interventions that strengthen leadership capacity and promote supportive organizational cultures can generate substantial improvements in patient satisfaction and institutional competitiveness. Tailored initiatives such as short-term workshops on adaptive decision-making and scenario planning, combined with cultural programs that encourage patient feedback, teamwork, and recognition of patient-centered practices represent feasible, low-cost strategies for resource-constrained settings like Addis Ababa.

## 8. Limitations recommendations for future research

Despite its contributions, this study has several limitations. First, the reliance on patient self-reports to measure service quality introduces the risk of response bias, as perceptions may be shaped by individual expectations or temporary experiences rather than objective indicators. Future research could address this by triangulating patient perceptions with staff perspectives or administrative data, such as wait times or clinical error rates, to provide a more holistic assessment of service quality.

Second, the cross-sectional design limits the ability to establish causal relationships between adaptive leadership, organizational culture, and patient-perceived service quality. Longitudinal studies would allow researchers to track how leadership behaviors and cultural dynamics influence service outcomes over time, thereby strengthening causal inference.

Third, the sample was confined to private hospitals in Addis Ababa. While this focus reflects the growing importance of the private healthcare sector in Ethiopia, it may limit generalizability to public institutions or other regions. Comparative studies across public and private hospitals, as well as across different low- and middle-income country (LMIC) contexts, could provide valuable insights into how healthcare environments shape the leadership culture service quality nexus.

Finally, the model did not account for other potentially influential factors, such as staff workload, organizational resources, or external policy environments, which may interact with leadership and culture in shaping service quality. Future research could incorporate additional mediators or moderators such as employee engagement, team dynamics, or resource availability and adopt mixed-method approaches to gain deeper insights into the mechanisms linking leadership practices with patient outcomes.
